# Endowing a NAO Robot With Practical Social-Touch Perception

**DOI:** 10.3389/frobt.2022.840335

**Published:** 2022-04-19

**Authors:** Rachael Bevill Burns, Hyosang Lee, Hasti Seifi, Robert Faulkner, Katherine J. Kuchenbecker

**Affiliations:** ^1^ Haptic Intelligence Department, Max Planck Institute for Intelligent Systems, Stuttgart, Germany; ^2^ Department of Electrical Engineering, Institute of Smart Sensors, University of Stuttgart, Stuttgart, Germany; ^3^ Department of Computer Science, Human-Centred Computing, University of Copenhagen, Copenhagen, Denmark

**Keywords:** human-robot interaction, socially assistive robotics, social touch, affective touch, tactile sensors, gesture classification

## Abstract

Social touch is essential to everyday interactions, but current socially assistive robots have limited touch-perception capabilities. Rather than build entirely new robotic systems, we propose to augment existing rigid-bodied robots with an external touch-perception system. This practical approach can enable researchers and caregivers to continue to use robotic technology they have already purchased and learned about, but with a myriad of new social-touch interactions possible. This paper presents a low-cost, easy-to-build, soft tactile-perception system that we created for the NAO robot, as well as participants’ feedback on touching this system. We installed four of our fabric-and-foam-based resistive sensors on the curved surfaces of a NAO’s left arm, including its hand, lower arm, upper arm, and shoulder. Fifteen adults then performed five types of affective touch-communication gestures (hitting, poking, squeezing, stroking, and tickling) at two force intensities (gentle and energetic) on the four sensor locations; we share this dataset of four time-varying resistances, our sensor patterns, and a characterization of the sensors’ physical performance. After training, a gesture-classification algorithm based on a random forest identified the correct combined touch gesture and force intensity on windows of held-out test data with an average accuracy of 74.1%, which is more than eight times better than chance. Participants rated the sensor-equipped arm as pleasant to touch and liked the robot’s presence significantly more after touch interactions. Our promising results show that this type of tactile-perception system can detect necessary social-touch communication cues from users, can be tailored to a variety of robot body parts, and can provide HRI researchers with the tools needed to implement social touch in their own systems.

## 1 Introduction

Social touch is an integral aspect of our daily interactions with colleagues, friends, and family. We touch other people to gain attention, communicate needs, and build empathy and attachment (Cascio et al., 2012; Van Erp and Toet, 2015). Touch promotes social bonding and cognitive development in children (Cascio et al., 2019) and is essential for emotional well-being (Harlow and Zimmermann, 1959). Affective touch, or touch with an emotional component, is used to convey our emotions to others through nonverbal communication, such as a hug. Simultaneously, the field of socially assistive robotics (SAR) continues to grow, with robots serving as assistants (Kim et al., 2013; Fitter et al., 2020), caregivers (Bilyea et al., 2017; Ferretti et al., 2018; Logan et al., 2019), and companions (Shibata and Wada, 2011; Jeong et al., 2015; Sefidgar et al., 2015). We believe it is imperative that such robots increase in awareness and intelligence by developing an understanding of social touch. Other modalities, such as visual and auditory sensing, are useful in their own regard but cannot replace touch perception. Physical contact and changes in contact force are both hard to see, especially when a robot’s camera has low resolution, poor lighting, a field of view that does not include the contact, or a disadvantageous viewing angle. While audio can sometimes be used to infer touch, this approach works only in a very quiet and controlled environment, which cannot be expected for human-robot interaction. While present social and socially assistive robots may feature other advanced capabilities, they are simply not equipped with adequate touch-perception capabilities; if they have any touch sensing, it is typically limited to detecting either on/off contact or a simple force threshold, and only at key locations on the robot’s body (Wada and Shibata, 2007; Robins et al., 2010). There is a serious need in the field of human-robot interaction for a simple yet robust social-touch sensing solution.

Endowing socially assistive robots with tactile perception has long been a challenging task due to the complex and expensive nature of whole-body tactile sensing (Wada and Shibata, 2007; Dahiya et al., 2010; Schmitz et al., 2011; Wood et al., 2019). Unlike the soft and robust skin of mammals, traditional tactile sensors have been rigid and fragile (Argall and Billard, 2010; Dahiya et al., 2010). Recently introduced fabric-based tactile sensor designs are promising for social touch due to their soft texture, simple design, and robustness (Luo et al., 2017; Day et al., 2018; Lee et al., 2019).

**FIGURE 1:**
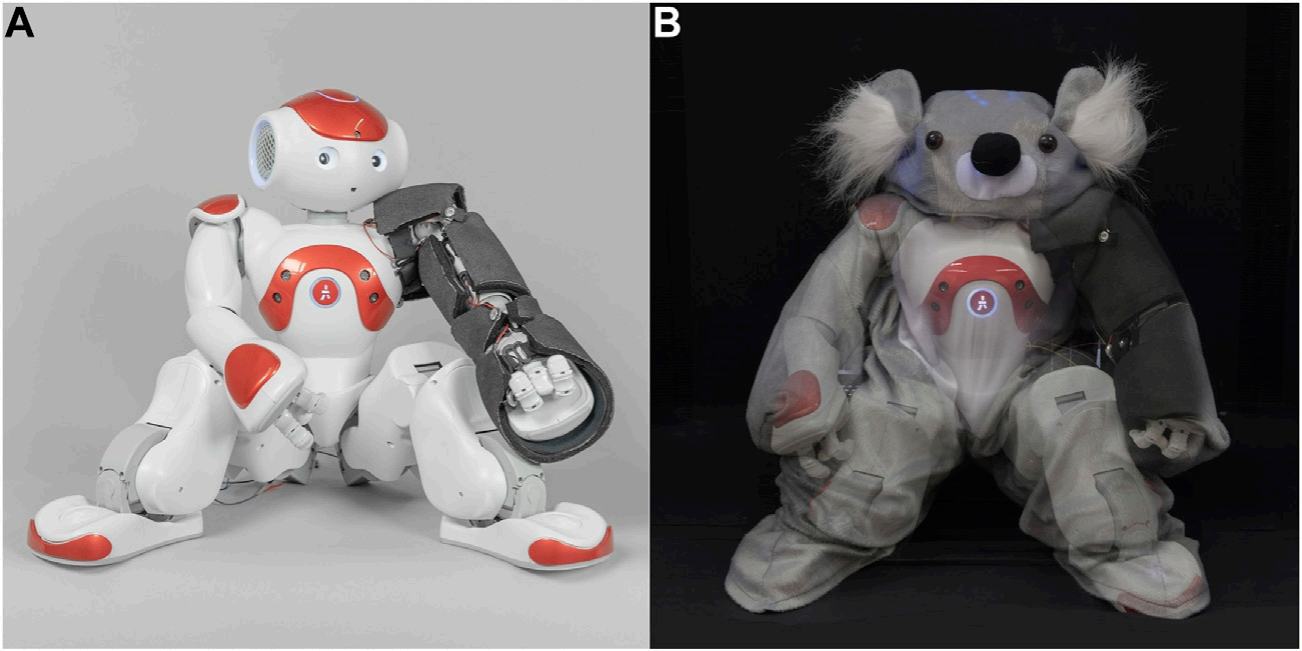
**(A)** Custom tactile sensors were built from fabric and foam to wrap around the four rigid segments of NAO’s arm. **(B)** As seen in this photo-merge, the sensors are secured on top of the robot’s plastic exterior and are hidden underneath a soft koala suit to create the robot companion HERA.

Even though existing social robots have limited tactile sensing, one does not need to wait and purchase an entirely new robot with built-in touch sensors to obtain whole-body tactile sensing that is robust and informative. Rather, an existing robot can be externally fitted with a tactile-perception system. This proposed approach can enable researchers, medical staff, teachers, and caregivers to continue to use technology they have already purchased and learned about, while adding a whole new channel of possible interaction methodologies alongside existing audio and visual sensing methods. We have created a robust tactile-perception system that is easy to manufacture, is pleasant to touch, and can be applied to existing rigid-bodied robots, as seen in Figure 1. It consists of fabric-based resistive tactile sensors whose outputs are processed using a touch-gesture classification algorithm. However, our goals go beyond providing a practical touch-perception solution; we also wanted to inform the creation of a whole touch-perceptive robot system and investigate users’ reactions to such a robot. We therefore added this tactile-perception system to a NAO robot to create our social robot prototype, the Haptic Empathetic Robot Animal (HERA). HERA is intended to eventually serve as a tool to help therapists teach children with autism about safe and appropriate touch (Burns et al., 2021b). While the NAO robot is frequently used in robot-assisted therapy studies to help children with autism (Tapus et al., 2012; Greczek et al., 2014; Suzuki and Lee, 2016), we intend to introduce practical touch-perception to add a new set of teaching opportunities. We tested the performance of this tactile-perception system on HERA in a user study with healthy adults.

Based on our findings, we present the following contributions:

• We created a design framework for **fabric-based resistive tactile sensors** that can be mounted on the curved surfaces of existing hard-bodied robots, with easy-to-follow step-by-step building instructions and an open-source database of four pre-made patterns.
• We share a **labeled dataset** of sensor readings and classifier source code from a user study in which fifteen adults touched the robot’s arm at each of the four sensor locations using five affective touch-communication gestures (hitting, poking, squeezing, stroking, and tickling) at two force intensities (gentle, energetic). After training on sensor data from the study participants, our touch-perception system perceives combined gesture and force intensity with an accuracy of 74.1% on held-out test data.
• We provide analysis of **participant impressions** of the overall robot system, including their reported feedback, their suggestions on how the robot should respond to the ten tested social-touch gestures, and our observations of their touch interactions with the system.
• We outline the **physical performance characteristics** of our tactile sensor design, including a robust sensing range from 0 to 30 N, gathered from sensors mounted to the hand, lower arm, upper arm, and shoulder of a NAO robot.

Before presenting our materials, methods, results, and discussion, we summarize relevant prior work in tactile perception for social-physical human-robot interaction (HRI), including touch-perception guidelines, tactile sensing, touch perception in previous robots, and gesture classification methods for social touch.

### 1.1 Touch-Perception Guidelines for Social Robots

While current social and socially assistive robots are generally limited in their touch-sensing capabilities, several teams have investigated whether touch perception is a worthwhile endeavour to pursue. They have also established which aspects of touch are most important for a robot to understand social touch communication from a human user and which features are less needed.

Tanaka et al. (2007) found that robots would benefit from having a system that can detect touch contacts across their whole body, and that contact locations can be grouped into general anatomical regions without fine spatial sensing resolution within each region. Experimenters coded videos from 45 field sessions of children interacting with the small humanoid robot QRIO in a classroom. The coders marked binary inputs indicating whether touch occurred across any of eight general body regions of the robot. By analyzing these data using a regression model, the researchers found that touch anywhere on the robot’s body was a strong predictor of the quality of social interaction, both between the human and the robot and between the human and their peers.

Andreasson et al. (2018) used video annotation to observe 32 adult participants performing 23 possible social-touch gestures on a NAO robot to convey nine different emotions. In order to replicate a human-human interaction study by Hertenstein et al. (2009), they visually divided the NAO robot’s body into 16 general body regions, and they recorded the gesture intensity, duration, general location, and touch type. The highest percentage of touches occurred on the robot’s arms and hands. In another part of the same user study, they covered the NAO in removable fabric garments intended to represent and eventually contain tactile sensors (Lowe et al., 2018). Their results showed that participants used stronger intensities, interacted for a longer duration, and touched more locations on the robot when it wore the garments compared to the uncovered NAO robot.

Burns et al. (2021a) recently investigated and established touch perception guidelines for a robot companion suitable for children with autism. The list was initially based on the literature and then refined through analysis of interviews with 11 experienced autism specialists. Since the guidelines were developed to fit individuals across a spectrum from touch-averse to touch-seeking, and the experts interviewed specialize in teaching safe and appropriate social touch, we believe their recommendations also largely cover the social-interaction needs of the general populace. The specialists wanted the robot to have a large tactile-sensing surface and did not ask for contact localization beyond general body regions. They further elaborated that the robot should sense both low-force and high-force contacts, and it should be inviting and pleasant to touch (e.g., friendly appearance and soft texture).

Based on these existing touch-perception observations and recommendations, we created a set of soft tactile sensors that are worn externally by a commercial robot and can detect the general body region of touch, touch type, and force intensity.

### 1.2 Tactile Sensing in Human-Robot Interaction

While tactile sensors have been studied for several decades (Dahiya et al., 2010), most development has been focused on robot fingertip sensors, which support object manipulation by providing high spatial resolution and accuracy across a small surface area (Argall and Billard, 2010). On the other hand, large-scale tactile sensors, which can provide sensing across the broad surfaces of a robot’s whole body, have received far less attention due to their high cost and high system complexity. For example, one existing approach is to utilize small discrete sensors that are spaced intermittently across the desired sensing area, such as the force-sensing resistors (FSRs) on the original Haptic Creature (Chang et al., 2010) or miniature capacitive force sensors like the SingleTact (PPS UK Limited, 2021). However, due to their small size (usually between 8 and 15 mm diameter), one must apply a great number of these sensors to cover the surface area of a robot. Such sensors are thus typically more costly per unit area than other methods, such as fabric-based tactile sensing, and they cannot sense contacts that occur in the regions between the sensors. Furthermore, this type of thin laminated sensor often experiences delamination, which harms the sensor’s performance (Tan and Tong, 2008); laminated flexible sensors can also be difficult to apply on a robot’s curved surfaces as they require a flat surface for good adhesion. However, recent advances in sensing materials and computation technologies have enabled the creation of some simple and affordable alternative large-scale sensing solutions (Luo et al., 2017; Teyssier et al., 2021).

Fabric-based tactile sensors are large-scale tactile sensors that are typically low cost and easy to manufacture. They are also flexible (Day et al., 2018), which makes them a better fit than rigid tactile sensors for curved surfaces like a robot’s arm. The simplest fabric-based design uses one layer of low-conductivity fabric sandwiched between two layers of high-conductivity fabric. When a user compresses the sensor, the two highly conductive layers come closer together, causing the sensor’s electrical resistance to decrease. However, due to the small distance (only one piece of fabric) between the two conductive layers, this design exhibits a low range of resistances in response to different force inputs. In the design of Day et al. (2018), an individual taxel demonstrates a dynamic force-sensing range from roughly 0.5–5.5 N. One way to increase the sensing range is to add a middle layer of plastic mesh, which serves as a spacer between the two conductive layers. However, an initial amount of force is required before contact can be detected through the plastic mesh. When indented with a 5 cm^2^ probe tip, the fabric sensor of Büscher et al. (2015) does not begin changing resistance until a threshold of roughly 2.5 kPa, or 1.25 N; gentle forces below this level are critical for perceiving affective touch (Taneja et al., 2019). While fabric-based sensors are cost effective and easy to make, this field lacks a sensor design that has both a *wide sensing range* and *good low-force touch detection*.

Our sensor design utilizes a center layer of low-conductivity foam rather than low-conductivity fabric with or without a plastic mesh. Using foam enables the sensor to detect gentle touches that would be blocked out by a mesh layer, while still having a wide sensing range. We test our sensor’s performance on a real NAO robot through both a user study and quantitative physical experiments.

### 1.3 Touch Perception in Socially Assistive Robotics

Most robots in SAR are very limited in terms of touch perception. The humanoid robots NAO and Pepper detect binary touch inputs – pressed or not pressed – through capacitive sensors at a small number of discrete key body locations. The baby seal robot PARO has touch sensors in its head and flippers, and it reacts positively to gentle touches and negatively to forceful touches (Wada and Shibata, 2007). While most of these systems are fairly simple, two examples of the state of the art for SAR touch perception are Kaspar (Wood et al., 2019) and the Haptic Creature (Sefidgar et al., 2015).

Kaspar is a child-like humanoid robot designed as an assistive tool for children with autism (Wood et al., 2019). The current iteration of Kaspar, K5, features fifteen force-sensing resistors (FSRs) positioned at discrete points across the hands, arms, feet, legs, chest and face of the robot. These FSRs are used to identify whether firm or gentle force is used on the robot (separating the levels at 0.6 N), but not what kind of gesture has been performed (Robins et al., 2010). In contrast to our goals, Kaspar’s FSR sensing areas are small, and its surfaces are rigid.

Envisioned as a therapeutic companion, the Haptic Creature is a furry lap-sized robot animal that calms participants by imitating slow breathing patterns (Chang et al., 2010; Sefidgar et al., 2015). The initial model of the Haptic Creature detected touch through 56 FSRs distributed across its body (Chang et al., 2010). In Sefidgar et al. (2015)’s later version, the FSRs were replaced with a large-scale fabric-based piezoresistive sensor array. The robot’s tactile sensing system was used to identify what gesture had been performed, but not to try to deduce the gesture’s force intensity. A random forest classifier with 20-fold cross validation was used to identify six gestures performed on the robot by ten participants with 88.6% accuracy (Cang et al., 2015); this performance is about five times higher than random chance. In contrast to our hardware goals, the Haptic Creature is a fully custom robot with a simple body and few degrees of freedom.

This paper is partially inspired by and aims to improve upon the touch-perception research conducted with Kaspar and the Haptic Creature. We provide soft tactile sensing coverage across an existing robot’s entire arm, rather than only at discrete points. We then use these sensors to create a tactile-perception system that simultaneously identifies both the force intensity and the type of gesture performed.

### 1.4 Gesture Classification Methods for Social Touch

To perceive the information gathered by tactile sensors as specific touch gestures, a robot needs a mental framework for understanding what the possible gestures are. This skill of tactile gesture recognition is often implemented by processing acquired tactile data through a machine-learning classifier that has been trained on labeled examples. These classification algorithms typically analyze the time-series touch data using data sampling windows with a fixed time duration (Cang et al., 2015).

To learn more about various classifier techniques, we refer the reader to the following papers, which detect social touch gestures using a variety of classification models: Bayesian classifiers (Jung et al., 2014), decision trees (Kim et al., 2010), LogitBoost (Silvera Tawil et al., 2012), neural networks (Stiehl and Breazeal, 2005), random forests (Altun and MacLean, 2015), and support vector machine (SVM) variations (Cooney et al., 2010; Jung et al., 2017). Additionally, Jung et al. (2017) provide a comprehensive list of research on the use of tactile sensing to detect and identify social touch, including a table identifying the surface touched (e.g., a mannequin arm, a stationary robot), the gestures performed, the classifier method, and the system’s classification accuracy.

The random forest classifier approach has been successfully utilized in several works studying touch gesture recognition (Altun and MacLean, 2015; Keshmiri et al., 2020), including the second version of the Haptic Creature (Cang et al., 2015). In a comparative study by Keshmiri et al. (2020), participants conducted gentle and strong versions of three touches on a mannequin wearing a vest with a 32 × 32 grid of pressure sensors: hitting on the chest, hitting on the shoulder, and hugging. The random forest classifier performed significantly better at identifying the different touches than five other classification approaches, achieving an average accuracy of 85% (about five times higher than the random chance level of 16.67%).

While many systems have classified social touch gestures, very few have sought to identify gesture and force intensity together. *Gesture type* and *force intensity* convey two distinct and important features of social touch (Burns et al., 2021a). By detecting both, a robot could potentially obtain a better understanding of the user’s intent and how to respond. The gesture-recognition algorithm we use to evaluate our tactile sensors is based on a random forest and classifies both gesture and force intensity.

## 2 Tactile Sensor Design and Fabrication

### 2.1 Sensor Overview

Our fabric-based tactile sensor design consists of a layer of low-conductivity foam sandwiched between two outer layers of high-conductivity fabric, which serve as electrodes. The two conductive-fabric electrodes are connected to a microcontroller (Uno, Arduino, Italy) via metal clothing snaps and wires. The sensor is powered by enabling the internal pull-up resistor of an analog input pin on the Arduino. One electrode layer is connected to this analog input pin, and the other electrode is connected to ground. This simple circuit creates a voltage divider formed by the internal pull-up resistor and our sensor; we measure the voltage drop across the fabric sensor to calculate its instantaneous resistance.

An Arduino’s internal pull-up resistors have resistances between 20 and 50 kΩ (Arduino, 2018). We tested the pins of our microcontroller and found them to have internal pull-up resistance values of approximately 37 kΩ. The chosen high-conductivity fabric (Shieldex CombiTex, Statex, Germany) has a sheet resistance of <1 Ω/sq., meaning that a square piece of material of any size would have an edge-to-edge resistance of 1 Ω, which is negligible. The chosen low-conductivity foam (6.0 mm RS Pro Low Density ESD Foam, RS Components, United Kingdom) has a sheet resistance that depends on the force exerted upon it; when undeformed, it is approximately 100 kΩ/sq., and it can decrease down to 0.1 kΩ/sq. when heavily compressed, giving the sensor a correspondingly wide sensing range. The electrical layers are placed on top of a base layer of nonconductive neoprene foam (1.5 mm, Neopren Solution GmbH, Germany) to disperse touch across a wider surface area and increase the softness of the robot form. Each layer is adhered to its adjacent layers at the edges using heat-activated tape (Thermal Bonding Film 583, 3M, United States).

Because of its uniform structure, applying the same contact at different places on this sensor produces approximately the same signal. Therefore, a single sensor equates to one tactile pixel (“taxel”) and does not provide location within the sensor or have multi-point touch discrimination. Identifying a general contact region of the robot’s body meets the guidelines for detecting social touch discussed in Section 1.1. Thus, this sensor design provides touch data that is both physically and computationally simple yet effective for social interaction. If an application requires more precise location information, such as splitting the upper arm into front and back halves, then multiple smaller sensors can be built and mounted next to one another.

### 2.2 Fabrication Process for Curved Surfaces

While a fabric sensor manufactured on a flat surface has good electrical performance on a flat surface, its performance significantly degrades when it is bent around a curved surface. This degradation occurs because all the layers of a flat-manufactured sensor are cut to the same size. When the sensor is applied to a curved surface, the outer layers of the flat-manufactured sensor must stretch to reach around the larger perimeter. Additionally, the innermost layers may also bunch up. These stretching and compressing deformations cause the sensor to perform as though it is already being touched, reducing its sensitivity and sensing range. This practical challenge is important to address because most rigid robot body parts are composed of complex shapes rather than flat surfaces; curved tactile sensors are thus required. After trying various mitigation techniques, we discovered that one can best solve this problem by *creating a curved sensor from the beginning*, using a curved building surface that closely matches the curvature of the sensor’s final intended location. We design the sensor’s dimensions to increase with each layer so that the material does not need to stretch.

**FIGURE 2:**
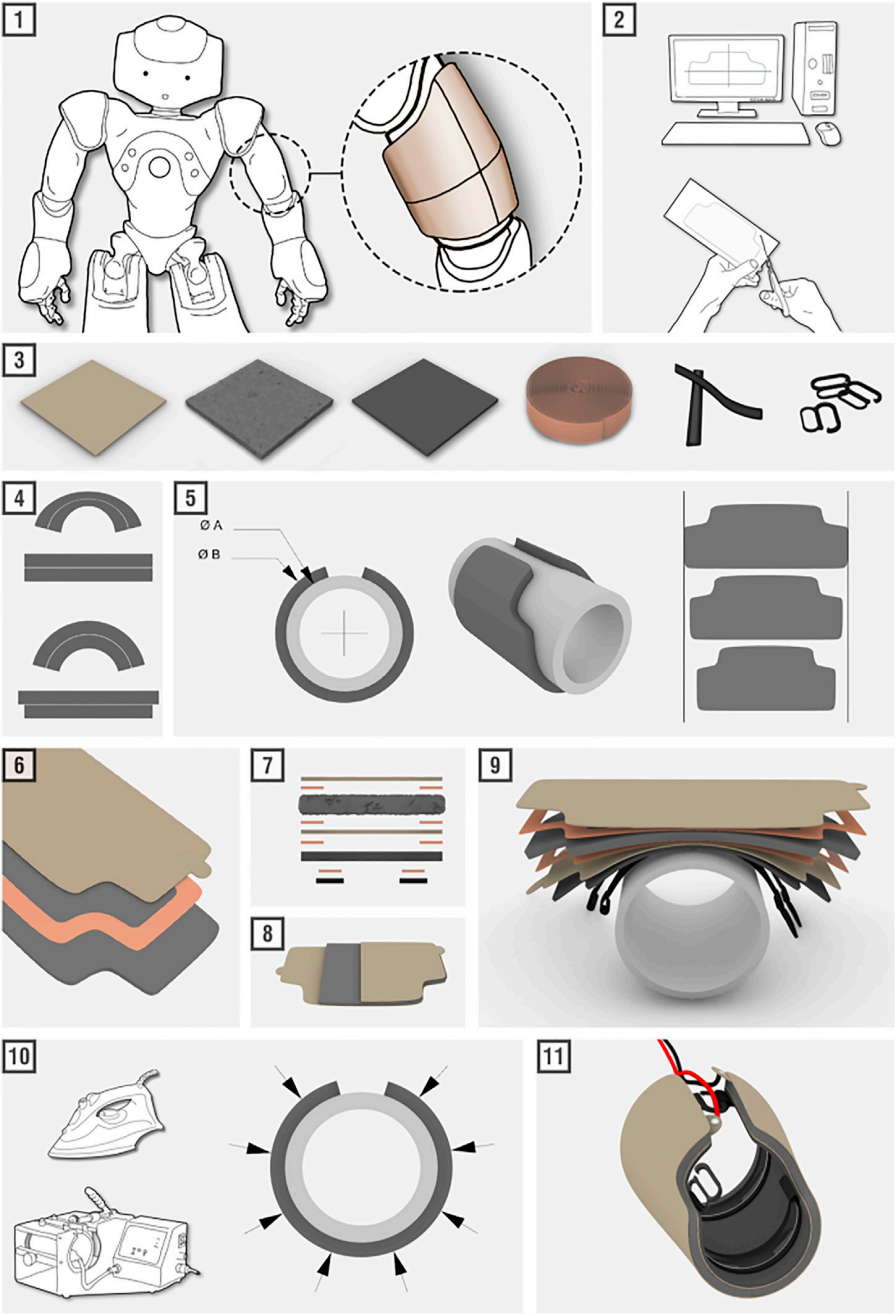
A visual guide highlighting the eleven key steps required for building our fabric-based tactile sensors.

We provide a full step-by-step visual guide of how curved sensors are constructed in Figure 2. We also provide a supplemental video showing the entire fabrication process, which can be summarized as follows. In **step 1**, we identify the shape and curvature of the sensor’s final location. We created the base layer of each sensor using tailoring: measuring the area by hand and drawing the resulting shape. One could also generate the base layer using methods such as 3D scanning or molding. In **step 2**, we create templates for the sensor’s subsequent layers, which can be determined either by sequentially placing and tracing each new layer or by using drafting software. In **step 3**, we assemble all of the necessary raw materials: high-conductivity fabric, low-conductivity foam, neoprene foam, heat-activated tape, rubber silicone elastic bands, and plastic clips. **Step 4** and **step 5** illustrate the phenomenon explained previously; for curved surfaces, the sensor layers must increase in size to compensate for the increased circumference that comes with increased layer height. In **step 6**, the materials are cut to the desired shape using scissors or a laser cutter. A tab-shaped protrusion is left on the end of each conductive outer layer. The tape is cut to 10 mm wide and lengths suitable for placement along all edges. **Step 7** shows the order of all material layers as a reference. Additionally, **step 8** indicates that the two tabs should be placed in different locations to prevent them from contacting, which would short the sensor. For **step 9**, we secure the sensor around a cylindrical form with a radius that closely matches that of the final sensor location on the robot. A plastic clasp is sewn onto each rubber silicone elastic band. The silicone bands are secured onto the Neoprene base layer using either sewing or heat-activated tape. The silicone coating increases the friction between the sensor and the outer surface of the robot, to keep the sensors stationary during interactions. In **step 10**, heat and pressure are applied to fuse the heat-activated tape, using methods such as a clothing iron or a mug press. For this study, approximately 149°C heat was applied using a clothing iron for roughly 3 s in each area [as recommended by the tape’s bonding guidelines (3M – Electronics Materials Solutions Division, 2015)], using a protective cloth covering and small circular movements to slowly work across the sensor. Each layer is added and heated incrementally to ensure the layers adhere properly. Finally, in **step 11**, the supply voltage and ground wires are connected to their respective electrode tabs via clothing snaps, and the sensor is ready to be mounted on the robot. The snaps also allow the sensor to be easily removed if needed, e.g., for repair or cleaning the setup. The final sensor is 10 mm thick.

### 2.3 Tailoring Sensors Across NAO’s Arm

As a proof of concept for our system, we built four sensors to cover the left arm of a NAO robot. The arm has four rigid segments, as seen in Figure 1 and Step 1 of Figure 2: the hand, the lower arm (between the wrist and elbow), the upper arm (between the elbow and shoulder joints), and the shoulder. Each sensor was fabricated around a glass or metal cylinder of a curvature matching its corresponding arm segment. The cylindrical radii for the hand, lower arm, upper arm, and shoulder were measured at roughly 3.5, 2.5, 3.0, and 4.0 cm, respectively. Rather than being a perfect cylinder, each arm segment has unique geometry, such as a bulge on the upper arm representing a bicep muscle, and a deep cavity in the hand to leave room for fingers. Therefore, each sensor was fabricated with a custom pattern for an optimal fit. We have publicly shared the patterns for creating these four sensors in an online repository (Burns et al., 2022a).

### 2.4 Estimated Costs and Fabrication Time

We purchased the high-conductivity fabric, low-conductivity foam, and neoprene foam in pieces that were 100 cm by 130, 100, and 55 cm wide, respectively. With these fabric quantities and the heat-activated tape, we estimate the price of the sensor’s raw materials to be 1.88€/10 cm^2^. As the surface area of the sensor increases, the percentage of surface area covered by tape will decrease, and so this price ratio will also decrease. The plastic clasps, silicone bands, and metal clothing snaps add a nominal cost. A sensor for the NAO’s upper arm, like that in Figure 2, with a surface area of 162 cm^2^ and a perimeter of 55 cm, would cost approximately 7.78€. The Arduino Uno microcontroller we used retails at roughly 20€.

We estimate that a novice maker using our sensor patterns would need approximately 2 h to create a fully operational sensor using scissors and a clothing iron. Using tools such as a laser cutter and a mug press should streamline this process to take closer to 45 min. Regardless of the tools used, the time needed per sensor would decrease if the maker prepares layers for multiple sensors in bulk, and as they gain experience with the techniques and materials. Beneficially, using the fabrication process and materials we propose, no waiting time is required between steps.

## 3 User Study Testing

After designing and constructing sensors for the NAO, we conducted a user study to learn about the performance of our tactile sensors as well as how users would receive a touch-sensitive robot system of the envisioned type. During initial testing of the sensor design, we observed that different touch gestures and force intensities could often be distinguished through visual inspection of the resistance data alone. We suspected that a machine-learning algorithm could automatically capture these patterns to perform accurate identification. Therefore, we hypothesized that the simple yet practical time-series resistance data produced using our tactile sensors would be sufficient to identify which sensor was being touched, the level of force intensity a user was exerting, and which gesture was being performed. Furthermore, our sensor design was motivated by the touch-perception guidelines detailed in Section 1.1. Using conductive foam not only gives our sensors a wide sensing range but also provides a soft and squishy feel. As Burns et al. (2021a) recommended a robot’s exterior to have such tactile qualities in order to promote user interaction, we also hypothesized that users would find our fabric-based sensors pleasant to touch and the act of touching a robot to be engaging and appealing.

To test these hypotheses, we introduced participants to HERA, a robot companion composed of a NAO robot wearing our tactile sensors and a koala costume (Burns et al., 2021b). We asked 15 adults to conduct five prominent social-touch communication gestures [as identified by Burns et al. (2021a)] at two force intensities on each of the four tactile sensors across HERA’s arm in a full-factorial design. To evaluate the usability of our sensors, we wrote a machine-learning-based gesture-classification algorithm that we trained to process the sensor data over time. The raw sensor data from our user study is publicly available online, along with start and end indices for all gesture performances, a MATLAB script for generating the indices, and the source code for our gesture-classification algorithm (Burns et al., 2022b). To evaluate the personal experiences of the users, participants answered several questions about their interaction with the robot through opening and closing evaluations with sliding-scale ratings and open-ended questions.

### 3.1 Participants

As our country was implementing lockdown restrictions due to the COVID-19 pandemic at the time of this study, we focused recruitment on our own research institute, including employees not in research positions and family and friends of employees. We distributed an institute-wide email advertisement.

We recruited 15 participants (7 female, 8 male) who were all adults (mean: 32, SD: 5), spoke English capably, and came from 10 different home countries, as reported in a demographic questionnaire. We also ran one pilot subject whose data are not included because the study setup and procedures were slightly modified after their session; participant numbers thus range from P2 to P16. Participants rated their familiarity with robots on a five-point scale. Two participants had no prior experience with robots, one had seen some commercial robots (novice), one had interacted with some commercial robots (beginner), seven had done some designing, building, and/or programming of robots (intermediate), and four had frequently designed, built, and/or programmed robots (expert).

### 3.2 Experimental Setup

To start, we secured the four custom-tailored tactile sensors across the NAO’s left arm. Next, a koala suit outer layer was secured on top of the sensors. The purpose of the koala suit is four-fold: to create a friendly robot animal appearance in order to invite touch [as recommended in Burns et al. (2021a)] to hide the added sensors, to further soften contact with the robot, and to serve as an additional electrical insulator to keep the user physically separated from the sensor circuit. We cut a hole in the suit behind the robot’s head and its internal cooling fan to prevent overheating. The wires that attach to the electrode tabs of each sensor were hidden underneath each sensor, guided up the robot’s arm, down the back, and out of the koala suit through a small opening. The wires connected to the Arduino, which was hidden behind a black tri-fold poster that stood on the table and behind the robot. A webcam was placed near the setup to record both the robot and the participants’ hands as they interacted with the robot. This video provided a ground-truth record of the testing to which the experimenters could later return if there were any irregularities in the data. The robot was powered on, and its arm motors were engaged to hold the same joint configuration with maximum stiffness for each test.

### 3.3 Procedure

**FIGURE 3:**
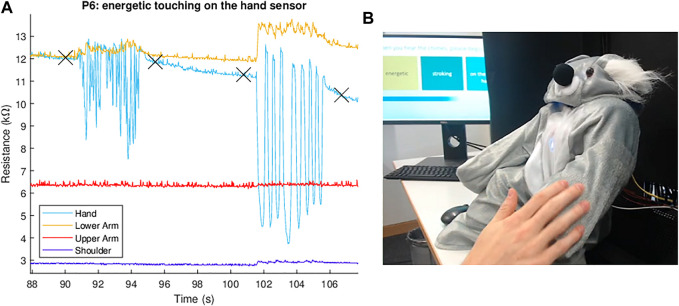
Real-time sensor readings show how the four sensor resistance values change **(A)** as the participant interacts with the robot **(B)**. The four × symbols on the plot mark the experimenter’s timestamps. In this example, the participant performed an energetic tickling gesture on the robot’s hand between 90 and 96 s and performed energetic stroking on the same body part between 101 and 107 s.

This user study was approved by the Ethics Council of the Max Planck Society under the Haptic Intelligence Department’s framework agreement as protocol number F008A. Participants not employed by our institution were compensated at a nominal hourly rate. Throughout the user study, the robot did not respond to any of the performed gestures; it simply held its constant pose. The four stages of the study proceeded as follows:

**1) Demographic questionnaire and opening evaluation (10 min)** – After providing informed consent, the participant filled out a demographic questionnaire that included topics such as country and familiarity with robots. The participant then completed an opening evaluation wherein they rated and commented on the tactile appeal of the robot’s two arms (with and without the added sensors) and also stated their level of agreement with seven general statements about the robot; these statements were adapted from a survey deployed by Fitter et al. (2020) and can be seen in Table 1.
**2) Instructions for touch interactions (5 min)** – The experimenter showed instructional slides explaining the upcoming touch interactions. The participant was introduced to the five gestures to perform in the study – “hitting”, “poking”, “stroking”, “squeezing”, and “tickling.” We also provided printed definitions for the touch gestures as defined by Yohanan and MacLean (2012) in their touch dictionary. Next, the participant was informed which force intensity they should use on the robot first – “gentle” or “energetic” – and practiced at least one sample trial of their first force condition.
**3) Touch interactions on the robot (2 × 10 min)** – The experimenter switched to an automated presentation that indicated the force intensity, sensor location, and touch gesture to use in each trial. Figure 3 shows sensor data being recorded as a participant interacts with the sensor; the automated instructions can be seen in the background of the inset image. The participant was instructed to use the same force intensity for all of the touch interactions in this half of the task. Additionally, gestures were performed on one sensor location at a time. At each sensor location, the participant executed each of the five gestures in a randomly assigned order. Each gesture was performed for 5 seconds, with a 5-s break of no touching between successive gestures. This approach is consistent with previous literature, such as Cang et al. (2015). Within the 5 seconds for touching, the users could do as many instances of the gesture as they wanted. This process was repeated twice for a total of three trials of each gesture. Once all of the gestures had been performed three times at the sensor location, the participant was instructed to move on to the next randomly ordered sensor location. This process was repeated until gesture data had been collected at all four sensor locations using the first force intensity. Finally, this entire step was repeated using the second force intensity. In total, each of the 15 users performed the two force intensities × 5 gestures × 4 sensors × 3 repetitions = 120 trials. We provide a supplemental video that shows a sample recording of the experimenter’s interface during this phase of the study.
**4) Closing evaluation (5 min)** – The participant rated their level of agreement with the same seven statements from the opening evaluation and provided any final comments. They were additionally asked how they expected the robot would react to the gestures and force intensities from the touch interactions.

**TABLE 1:** The seven questions in the robot acceptance survey asked in both the opening and closing evaluations.

I like the **presence** of the robot
I feel **threatened** by the robot
I am **afraid** of the robot
I am afraid to **break** something while using the robot
I think using the robot is a **good** idea
I feel **safe** touching the robot
It could be **useful** to touch the robot

The resistance measurements from all four sensors were recorded at a sampling rate of 40 Hz during all gesture performances. The experimenter held a manual button that was connected to the microcontroller. For every trial, the experimenter pressed this button just before the participant began a gesture and again immediately after the gesture’s completion, which created timestamps marking the boundaries of each gesture performance.

### 3.4 Gesture Classification

The resulting raw data from the four tactile sensors were then prepared for classification. As the randomized gesture order was generated in advance, the experimenter had a ground-truth order with which to pair the timestamps. We could then systematically label gesture data for each dataset; time spans with no gesture were labeled “none.” Every dataset was inspected in Tableau Software to ensure there were no missing or added markers, which would cause mislabeled data. Upon detailed inspection, we found that momentary electrical shorts to ground had occurred in the shoulder sensor’s resistance data during some gesture performances. Most of these shorts occurred during participants’ interactions with the neighboring upper arm sensor. We also discovered two trials where the hand and lower arm sensor temporarily shifted to identical resistance values, which we assume was caused by momentary electrical contact. We discarded all trials that contained one or more momentary shorts to avoid providing corrupted data to the classification algorithm; in total, 117 of the 1800 recorded gesture performances (15 participants × 120 trials per participant) were removed, leaving 1683 labeled intervals.

Next, we developed and refined the gesture classification algorithm. The classification was conducted using Python and the Scikit-learn library. We segmented the time-series data using a moving window size of *w* data points and an overlap size of 0.5*w* between successive windows. If a data segment had an overlap of at least 0.75*w* with any of the gesture time spans, the segment was labeled with that gesture. Otherwise, it was labeled as “none.” For each segment, we calculated a vector of 124 features (31 from each of the four sensors), including the entropy of each resistance signal, and the sum, max, min, average, median, standard deviation, variance, area under the curve, interquartile range, and number of peaks for the signal itself, as well as all ten of these metrics for the signal’s first and second derivatives over time. These features were selected based on previous work on touch gesture classification, such as Gaus et al. (2015).

We trained and tested a random forest classifier on the raw data segments using a 70%–30% train-test scheme that was randomly sampled across all participant data. We selected a window size of *w* = 80 samples (about 2 s), as it provides good test accuracy on gesture classification and a reasonably fast reaction time for future responses from the robot. This window size is also consistent with prior work on touch gesture classification, such as Cang et al. (2015). The parameters we used for the random forest model can be found in Table 2.

**TABLE 2:** The parameter values that were used for the gesture-classification algorithms. Unless otherwise specified, the same value was used for classifying contact location, intensity, gesture, and gesture and intensity together.

Parameter	Value
bootstrap	True
class_weight	None
criterion	“gini”
max_depth	Location: 40
	Intensity: 20
	Gesture: 40
	Gesture and Intensity: 32
max_features	20
max_leaf_nodes	None
min_impurity_decrease	0.0
min_impurity_split	None
min_samples_leaf	1
min_samples_split	8
min_weight_fraction_leaf	0.0
n_estimators	Location: 50
	Intensity: 100
	Gesture: 100
	Gesture and Intensity: 100
n_jobs	1
oob_score	False
random_state	None
verbose	0
warm_start	False

## 4 User Study Results

This section summarizes the results from our user study, including the gesture classification algorithm’s performance, the participants’ answers from both the opening and closing evaluations, and our observations of notable unexpected behaviors that users performed while interacting with the robot.

### 4.1 Classification Results: Location, Force, and Gesture

**FIGURE 4:**
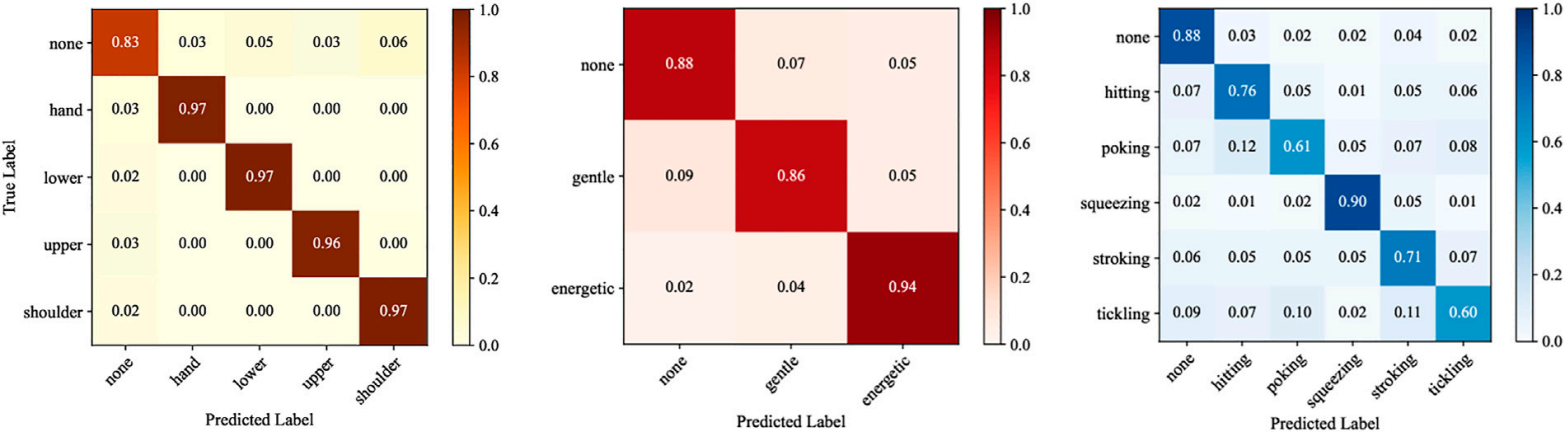
Normalized confusion matrices showing the classification results for location, force intensity, and gesture.

In all cases, the classification algorithm operated on the simultaneous data from all four sensor channels. To characterize the expected performance in unstructured interactions, we also evaluated the system’s ability to recognize the lack of contact, which was labeled “none.” Figure 4 shows the gesture classification algorithm’s performance as confusion matrices, classifying location, force intensity, and gesture individually. The location of each touch interaction was identified with 96% or greater accuracy for all sensor locations (chance level is 1/5 = 20%). The system correctly identified force intensity with an average accuracy of 89.3% (chance level is 1/3 = 33.3%). In the case of identifying only gesture, without intensity, the classification system had an average accuracy of 74.3% (chance level is 1/6 = 16.7%). We also looked at the gesture classification accuracy by sensor location: gestures on the hand, lower arm, upper arm, and shoulder had an average classification accuracy of 78, 74, 77, and 68%, respectively.

### 4.2 Classifying Force and Gesture Together

**FIGURE 5:**
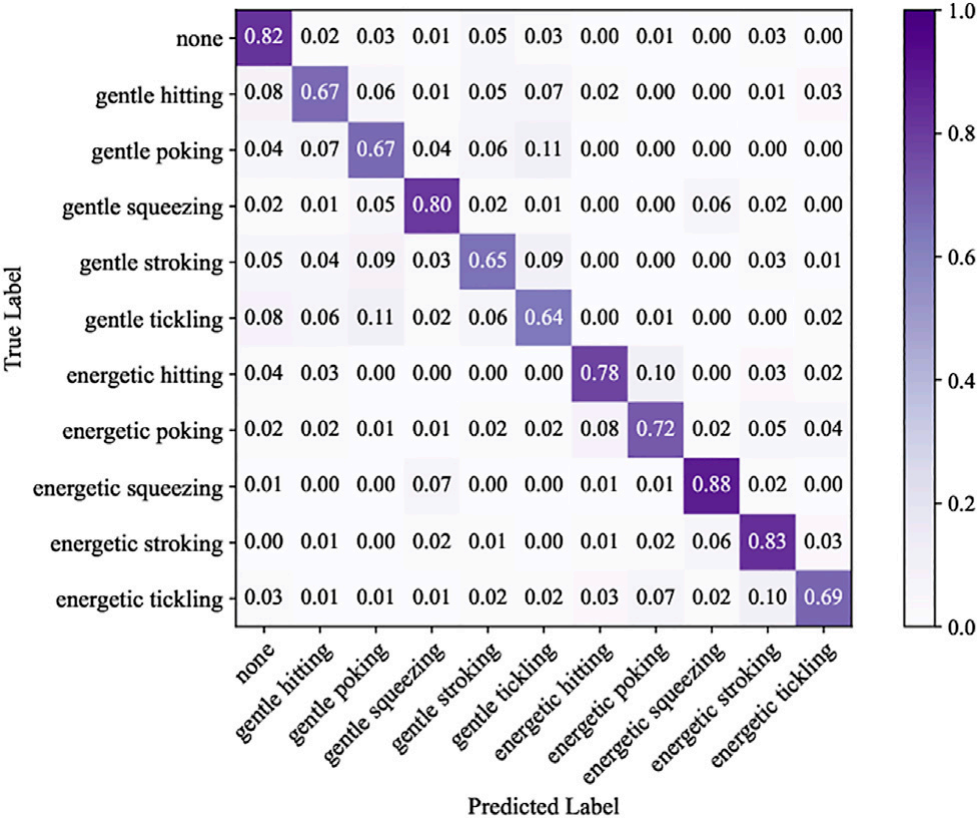
Normalized confusion matrices showing the classification results for both force intensity and gesture together.

Next, we present the case where the gesture classification system predicted both gesture and force intensity together. The resulting confusion matrix is presented in Figure 5. The system had an average label accuracy of 74.1%, which is about 8.2 times higher than the chance of randomly guessing a gesture correctly (1/11 = 9%). Energetic squeezing had the highest recognition rate at 88%. Gentle tickling was the hardest to identify, obtaining 64% recognition accuracy. Gentle tickling was most commonly confused with gentle poking (11% occurrence). Compared to results based on identifying gesture alone, identifying both gesture and force level improved the recognition accuracy for poking and tickling at both force levels. For the hitting and stroking gestures, the energetic force level had higher identification accuracy, and the gentle force level had lower identification accuracy. Finally, squeezing without a force level had a 90% recognition rate, whereas gentle squeezing and energetic squeezing had 80 and 88%, respectively.

### 4.3 Preference on the Feel of the Tactile Sensors

Participants were asked to rate the feel of both the robot’s left and right arms on a scale from 0 to 10, with 0 being “Unpleasant to touch” and 10 being “Pleasant to touch.” While both arms were covered with the soft koala suit, the robot’s left arm also had the added fabric-based tactile sensors underneath. One participant ranked both arms as equally pleasant, and all other participants ranked the arm with the added tactile sensors as more pleasant to touch. Anderson-Darling tests confirmed that participants’ ratings for both arms were normally distributed. A paired-samples *t*-test revealed that there was a significant difference in the pleasantness scores for the feel of the arm with the added tactile sensors (mean: 8.0, SD: 1.3) and the feel of the arm without the added sensors (mean: 3.5, SD: 1.7, t (14) = –6.5, p < 0.001).

Participants were also asked to elaborate on their numerical ratings with an open-ended written response. Several participants referred to the sensor-covered arm with positive phrases such as “soft and squishy”, “friendly”, “warm”, and “quite pleasant.” Conversely, the arm without added sensors was referred to with phrases such as “stiff” and “hard and unnatural.” Another participant wrote, “It would be even better if the whole robot was squishy.”

### 4.4 Perceptions Before and After Touch Interaction

**FIGURE 6:**
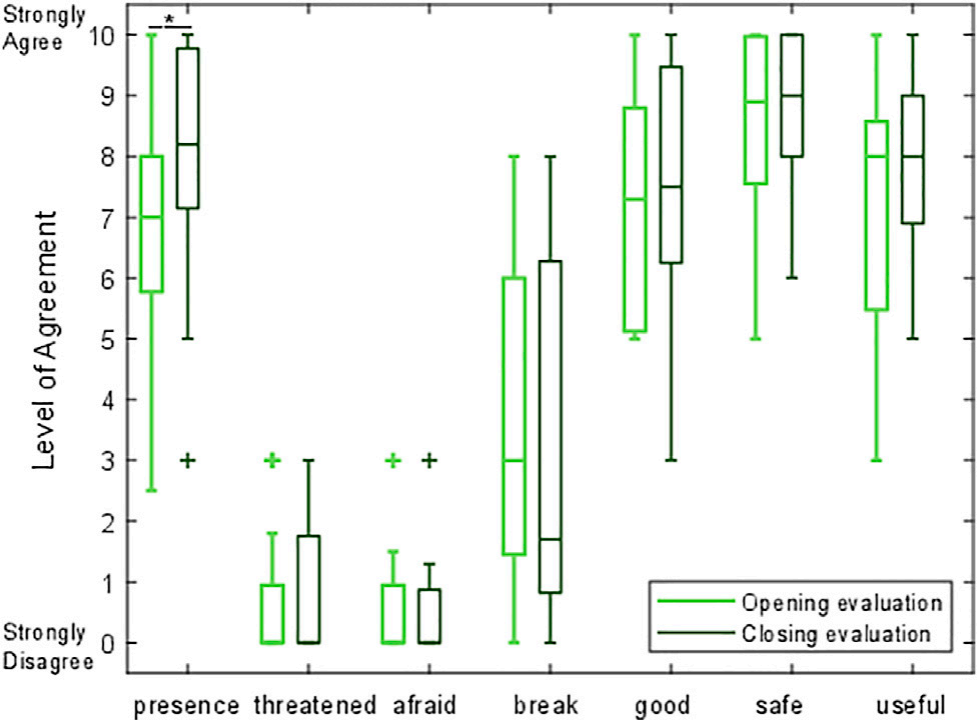
A box-and-whisker plot comparing participants’ level of agreement with seven questions before and after interacting with the robot. For each distribution, the central line indicates the median, the box shows the interquartile range (IQR), the whiskers show the range up to 1.5 times the IQR, and + marks indicate outliers.

Participants rated their agreement with seven statements (Table 1) in the opening and closing evaluations on a scale from 0 (“Strongly disagree”) to 10 (“Strongly agree”). The results can be seen in Figure 6, labeled by the bold keyword in each statement.

Anderson-Darling tests revealed that the assumption of normality was violated for the results of four opening evaluation statements (threatened, afraid, break, and safe), and for three of the closing evaluation statements (threatened, afraid, and safe). Therefore, we evaluated each of the seven robot acceptance statements with a Wilcoxon signed ranks test. We found that only one prompt underwent a statistically significant change in rating. Participants agreed with the statement, “I like the presence of the robot,” significantly more after the touch interaction sessions (mean: 7.9, SD: 2.0) compared to before the touch interactions (mean: 6.9, SD: 2.1, *n* = 15, Z = 2.937, *p* = 0.003). There were no other statistically significant changes in ratings among the robot acceptance questions. The non-significant results for the remaining comparisons are as follows: threatened (*n* = 15, Z = 0.677, *p* = 0.498), afraid (*n* = 15, Z = -0.405, *p* = 0.686), break (*n* = 15, Z = -1.099, *p* = 0.272), good (*n* = 15, Z = 1.482, *p* = 0.138), safe (*n* = 15, Z = 1.265, *p* = 0.206), and useful (*n* = 15, Z = 1.425, *p* = 0.154).

### 4.5 Expected Robot Reactions

**FIGURE 7:**
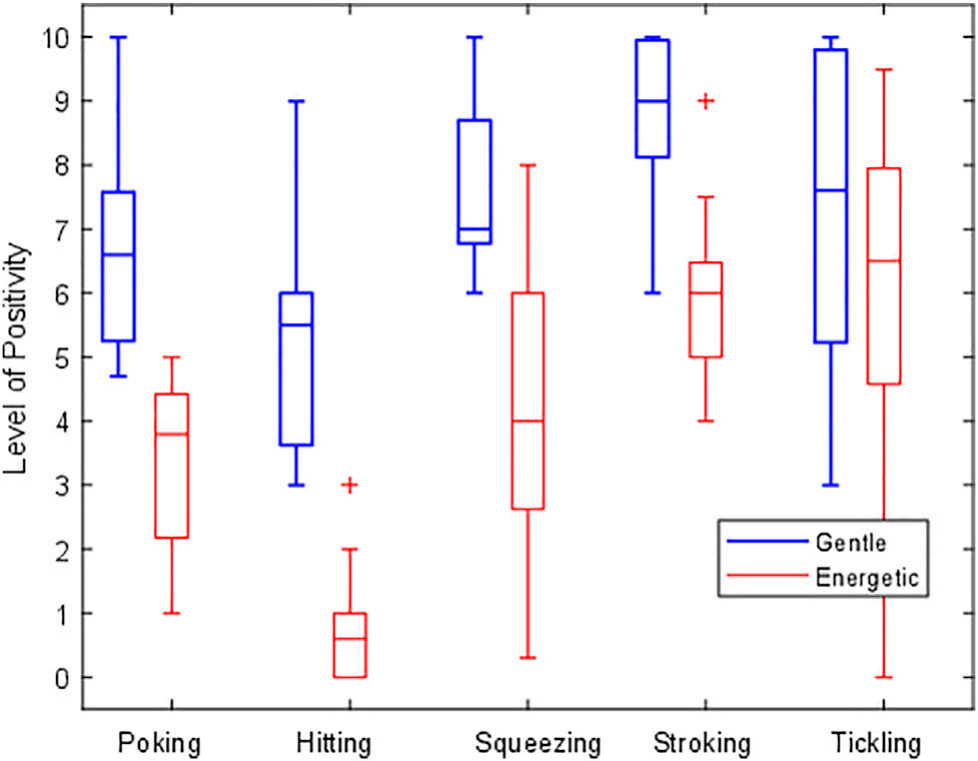
A box-and-whisker plot showcasing participants’ ratings of how they expect the robot would react in the future to the various touch gestures. A rating of 10 indicates a very positive response, and a rating of 0 indicates a very negative response. Touches with a gentle force intensity are shown in blue, and energetic gesture variations are colored red.

The participants rated how they expected the robot to react to the various touches, on a scale from 0 (a very negative reaction) to 10 (a very positive reaction). The resulting ratings can be seen in Figure 7 in the form of a box-and-whisker plot. Energetic poking, energetic hitting, and energetic squeezing all had median responses below 5, indicating that participants expected the robot to give a generally negative response for these three actions. All other gesture and force combinations had a median value higher than 5, signifying a generally positive response. However, it is interesting to note that some actions did not have a clear consensus between participant responses. For example, while energetic squeezing and energetic tickling received median ratings of 4.0 and 6.5, respectively, their relatively large interquartile ranges show that participants predicted both positive and negative responses. Some participants explained that they felt the response from the robot should be situation- or mood-dependent. For example, a deep squeeze could provide comfort and support, or it could be rough and hurtful.

### 4.6 Notable Observations

During the course of our study, some participants exhibited two unexpected behavioral patterns. We share these observations to provide additional context for the gesture classification and survey results.

**FIGURE 8:**
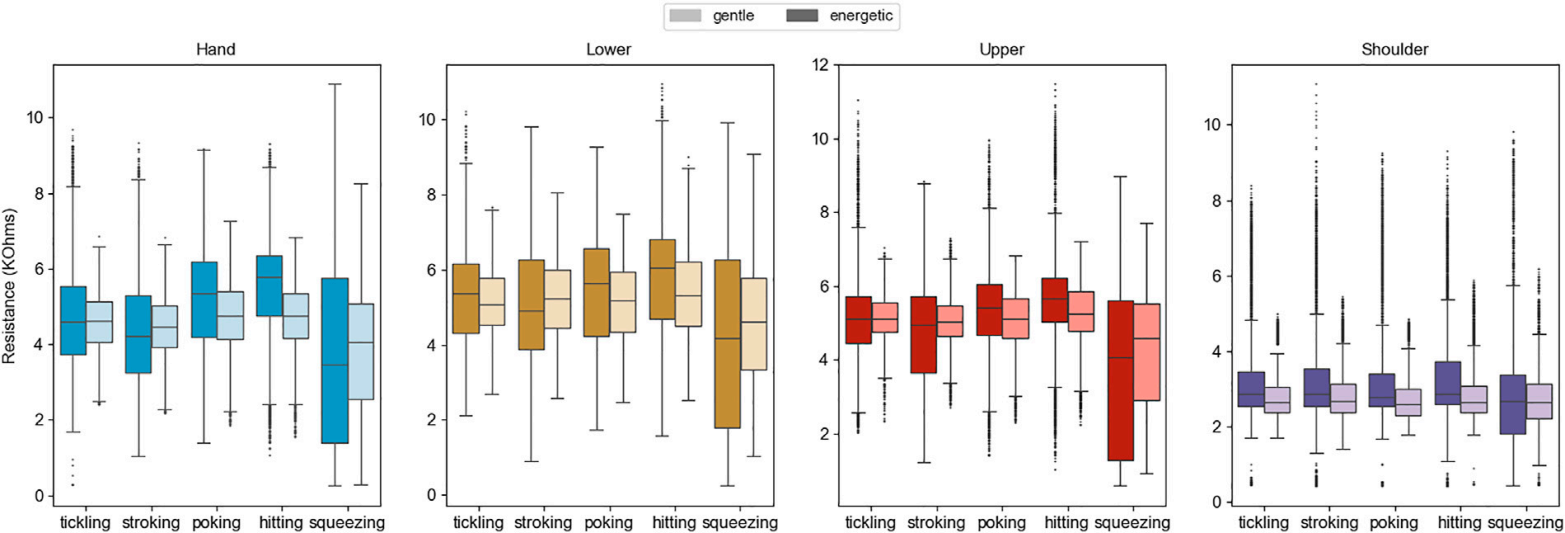
Box-and-whisker plots indicating the range of sensor resistance readings during all participants’ cumulative gesture performances. Gentle trials are shown in a pale color directly next to the more darkly colored energetic trials of the same type.

First, we observed that participants appeared to have differing perceptions of “gentle” and “energetic.” A gentle touch was typically reflected in the data as a narrow range of resistances, and an energetic contact tended to translate to a wide range. Figure 8 provides box-and-whisker plots grouped by sensor location to show the resistance ranges observed during each gesture. The same data are presented by participant in the supplementary materials for this manuscript. Although energetic performances typically did have higher ranges, some participants applied the same amount of force for both touch interaction sessions (e.g., P3 and P9). Additionally, energetic gestures by some participants were in the range of gentle gestures performed by others, such as P7’s unusually strong “gentle” gestures compared to P2’s low-range “energetic” gestures. We believe that individual preference, inattentiveness, and fatigue may have all played roles in generating these varying performances. Some participants were also hesitant to touch the robot energetically; for example, participant P4 wrote in the closing evaluation, “I felt uncomfortable with [performing] the energetic gestures as if it was a living creature.”

Additionally, of all the gestures we instructed participants to perform, we observed that the hitting gesture evoked the most vocal responses. Several participants expressed reluctance or distress at being instructed to hit the robot. During the touch interactions, one participant (P4) said, “I don’t like to hit the robot.” Another participant, P5, asked, “Do you want me to actually hit the robot?” While performing a set of energetic touch gestures, P6 hit the robot with particularly high force and then quickly said, “Oh, sorry!” to the robot.

## 5 Physical Sensor Testing and Results

While conducting the user study, we noted that the shoulder sensor appeared to have a smaller sensing range than its counterparts. Due to all four sensors’ differences in surface area, shape, and curvature, we were curious to understand whether and how their sensing performance varied.

### 5.1 Physical Experiment Procedure

Our physical sensor experiments utilized the same setup as the user study experiment, except that rather than touching the robot directly, the experimenter used a compression load cell (FC-22, TE Connectivity, Switzerland) attached to a cylindrical indenter tip with a diameter of 13 mm (to mimic the size of a fingertip).

We wanted to evaluate the fabrication and performance consistency of our sensors by testing the sensitivity at several points across each sensor’s surface area. We also wanted to characterize the hysteresis of our sensors – the change in sensor resistance output values based on whether the external force on the sensor is increasing (loading force) or decreasing (unloading force). To accomplish both of these goals, an experimenter used the indenter tip of the load cell to press across the surface area of each tactile sensor with a force range of 0–30 N. To determine whether the koala suit affects the system’s performance, this experiment was performed both with and without the koala suit on top of the sensors. Data collection always started from no contact. The experimenter pressed down with the load cell until it reached 30 N and then withdrew the indentation again back down to no contact. Each touch indentation occurred at intervals spaced roughly 2 cm apart across each sensor. The experimenter simultaneously provided support to NAO’s arm at the next closest arm segment to prevent damage to the robot’s fully engaged motors. The experimenter was careful not to touch the sensor currently being tested while supporting the robot’s arm.

### 5.2 Physical Experiment Results

**FIGURE 9:**
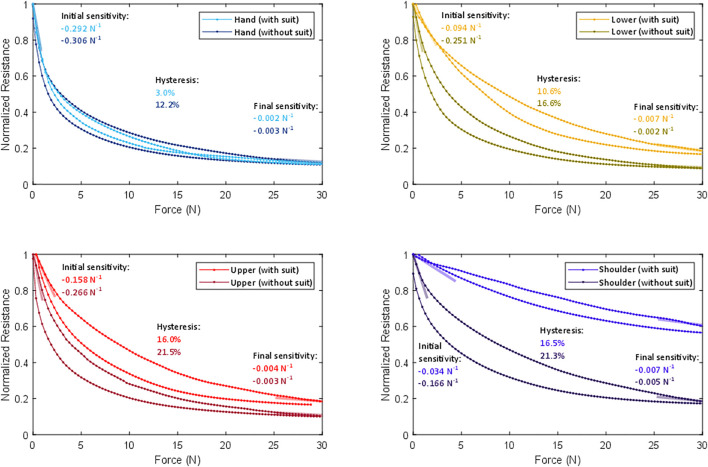
Normalized comparisons of individual sensor’s resistances across the tested force range with and without the koala suit, averaged across all trials of each physical experiment. The initial and final sensitivities are represented using semi-transparent lines oriented at the appropriate slopes. Each plot also lists the corresponding numerical values for sensitivity and hysteresis.

Performance markers were calculated for each of the four tactile sensors with and without the koala suit. First, it was found that the sensors have different initial resistances (i.e., the measured resistance when no external force is being applied) and different sensing ranges (i.e., the full range of resistance values a sensor may have depending on the force exerted upon it) due to their differing surface areas, manufacturing variations, and pre-compression by the koala suit. For example, with the koala suit on, the hand sensor had an average initial resistance of 11.5 kΩ, while the lower arm, upper arm, and shoulder had resistances of 18 kΩ, 9.5 kΩ, and 4.0 kΩ, respectively. Therefore, Figure 9 provides a normalized comparison of the measured resistance, *R*, by dividing each reading by that sensor’s initial resistance, *R*_0_, in that condition. This visualization shows that the sensors on the hand, lower arm, and upper arm demonstrate very similar sensing behavior relative to their starting resistance, as does the shoulder sensor in the “without suit” condition. The shoulder sensor shows much less response when in the koala suit. Looking more closely, one sees that the lower arm and upper arm sensors also become somewhat less sensitive when covered by the suit.

Figure 9 is annotated to show each sensor’s initial sensitivity, final sensitivity, and hysteresis, with and without the koala suit present. The initial and final sensitivities for each sensor were calculated with and without the suit using the slope of the corresponding force-resistance loading curve; we calculated the average slope from the first and last five data samples, respectively. With the suit, the average normalized initial sensitivity of the hand, lower arm, and upper arm sensors is –0.181 N^−1^. The average final sensitivity was –0.004 N^−1^, confirming that all three of these sensors reach their saturation point at roughly 30 N. With the suit, the shoulder sensor was found to be nearly 10 times less sensitive than the other three sensors at initial contact and similarly sensitive at final contact. Returning to the other three sensors, the human sense of touch prioritizes good resolution at low force and has coarser resolution at high force. For example, on the arm, one experiences a pleasant mood from affective touches occurring around 0.6 N (Taneja et al., 2019) and a maximum pain threshold at 25 N (Melia et al., 2015). Aside from the shoulder sensor, which will be discussed below, our sensor design emulates this nonlinear response, making it suitable for detecting a broad range of social-touch gestures.

The hand, lower arm, and upper arm sensors all showed fairly consistent initial sensitivities across their surface areas. We observed that they all demonstrated a slightly diminished initial sensitivity around the perimeter, potentially due to the layers of non-conductive heat-activated tape that bind the layers at the edges. The least sensitive point on any edge of these three sensors had an initial sensitivity registered at –0.044 N^−1^, which is about four times worse than the average in the center and twice as good as the average in the center of the shoulder sensor.

The effect of hysteresis is present and consistent across indentations; it appears as distinct loading and unloading curves in Figure 9. We numerically quantified the average observed hysteresis for each of these eight conditions by calculating the ratio between the maximum vertical separation between the loading and unloading resistances across the tested force range divided by the total range of the minimum and maximum resistance readings. Without the suit the four sensors show between 12.2 and 21.5% hysteresis. When the suit is added, the hysteresis decreases somewhat.

## 6 Discussion

This paper has presented a soft, low-cost, easy-to-manufacture tactile perception system that can be externally fitted onto existing hard-bodied robots for social-physical human-robot interaction. We sought to create a system that can work on curved surfaces and differentiate both the contact gesture and force intensity applied. Our system’s hardware consists of a set of pre-curved fabric-and-foam-based tactile sensors attached to the left arm of a NAO robot. We analyzed the outputs of these four sensors by applying a gesture-classification algorithm to a dataset collected during a user study and also performing select physical experiments. Furthermore, we investigated users’ reactions to interacting with a touch-perceiving robot through opening and closing surveys. This section reflects on the strengths and limitations of the scientific methods we utilized, and it discusses the contributions of our work toward future research in human-robot interaction.

### 6.1 The Shoulder Sensor

In the user study, the shoulder sensor had the lowest gesture classification accuracy of the four sensors (68 *versus* 78, 74, and 77%). The physical experiments showed that the shoulder sensor was nearly ten times less sensitive than the other three sensors. Why did this sensor perform so poorly? While we originally believed that the cause lay with our fabrication process, investigation revealed that the external koala suit was the core issue. We customized a child-size koala pajama suit that roughly fit the robot. However, unlike children, the NAO robot has large shoulder pads. Since we did not tailor the suit to provide additional room in the shoulders, the koala outfit pre-compressed the shoulder sensor. When we measured this sensor’s response with the suit on, we obtained an initial resistance that was much lower than without the suit; the compression tests with the suit were thus measuring only the tail-end of the force-resistance curve, after the sensor had already been greatly compressed. When the suit was removed and the shoulder sensor was measured separately, it performed similarly to the other three sensors. Therefore, if the robot is fitted with an external suit, it is important to ensure that the suit fits consistently across the sensor system. Moving forward, we will modify our koala suit so that it no longer exerts pressure onto the shoulder sensor in comparison to the other sensors.

### 6.2 Physical Design Implications

We discovered some aspects of our sensor design that should be addressed in future versions of similar tactile sensing systems. First, while the top and bottom faces of the sensors are covered with insulating material, the sides of the sensor are exposed. When the edges of two sensors touch, they electrically connect and interfere with each other’s signals. We had to remove 6.5% of the recorded gesture trials from our dataset due to different sensors experiencing momentary electrical shorts. The electrically exposed sensor edges could be better protected by adding an insulating layer that surrounds the entire sensor, though care should be taken not to reduce the active sensing area or introduce hard edges that disrupt the feel of the system (Burns et al., 2021a).

Secondly, we observed that the sensors display a peak of high resistance after contact and release, followed by a slow descent back to the baseline resistance, rather than an immediate return; an example of this behavior is visible in the hand sensor data in Figure 3. We suspect this slow return down to baseline resistance has mechanical origins. The sensor layers are secured together around the perimeter with heat-activated tape, leaving the middle of the sensor to have a small air gap between layers, which helps the sensors detect low-force touches. However, when the sensor is touched and released, the foam layer expands more slowly than the outer fabric layer. As the foam fills in to its original size, the air gap between these two layers is reduced, increasing the conductive surface area back to its original baseline. This phenomenon causes the sensor resistance to have somewhat different values depending on how recently it was touched. In the future, this variable air gap could be removed by using conductive adhesive across the whole surface area of each sensor, rather than just taping the edges. However, this solution may also lower the sensor’s ability to detect low-force touches.

Incidentally, we also found that the baseline sensor resistance gradually increases over the course of months, presumably due to tarnishing of the conductive fabric and foam; normalizing by the sensor’s initial resistance in a session removes this effect. Finally, even though we fixed the sensors in place with clasps and the silicone-grip bands, we found that sensor locations slightly shifted during the user study when the participant touched the robot particularly energetically. Moving to slightly different poses on the robot’s body changes the baseline resistance of these sensors, as it changes their curvature; an example of this behavior is visible in the lower arm sensor data in Figure 3 at the start of the second gesture. We tried applying a high-pass filter at 0.5 Hz to remove the resulting baseline shifts from our user study dataset and also stabilize the slow return to baseline mentioned above. However, we ultimately omitted this processing step as we found it did not improve the classification accuracy, most likely because it also discards useful information about the intensity of contacts that occur. Instead, these changes can be reduced in the future by securing the sensors more tightly in place on the robot’s body parts, either by using higher-friction straps that can be tightened, or by securing the sensors with another mechanism such as double-sided tape.

### 6.3 Taxel Size

As each of our sensors acts as a single taxel, we identified the general touch location based on where the sensor was fastened on the robot’s body. It is possible that attaching several smaller sensors to each rigid body part could improve gesture recognition accuracy, since some gestures tend to move across the surface. However, increasing the number of taxels would also increase the hardware complexity and computational load of the touch-perception system. Low system complexity is an important design feature when one wants to add sensors to a large portion of the robot body, as we plan to do in the future. Furthermore, the borders between neighboring sensors might be particularly susceptible to electrical shorting and could diminish the system’s pleasant feel. While we showcased the quality of gesture classification that is possible using four sensors that each cover a relatively large surface area, future researchers will need to consider the size and number of taxels best suited for their intended application.

### 6.4 User Study Limitations

The majority of our study participants had intermediate- or expert-level familiarity with robots (7 and 4 out of 15, respectively). While we had hoped to gather data from more novices, the COVID-19 pandemic limited our recruitment to participants with some connection to our research institute. Having participants who are less experienced with robots may have produced different results. However, recruiting internally enabled us to conduct this preliminary user study despite pandemic restrictions, which was essential for evaluating our tactile sensors and understanding participants’ perceptions of the overall robot system. Furthermore, the users of future touch-perception systems for robots are most likely to already be familiar with robots. Additionally, there may have been some self-selection bias in our recruitment, with only those positively inclined toward touching robots answering our email. Nonetheless, our participants were diverse in many ways, such as home country, gender, and age, and they voiced a wide range of opinions about the presented robotic system.

We gave our participants limited instructions during the touch interaction sessions. By not providing specific numerical ranges for the force intensities to exert, we encountered a wide range of perceptions of what it meant to perform gestures gently or energetically. Furthermore, in the case of the “hitting” gesture, two participants actually conducted a “poking” gesture instead, including a participant who said they did not enjoy hitting the robot. Such behaviors make the classification problem nearly impossible and lead to some misclassifications. However, we believe that using these limited instructions enabled us to record participants contacting the robot very naturally, creating a more general classification model that we believe will be useful beyond this user study during everyday interaction.

### 6.5 Visual Appearance of the Robot

Our robot HERA has a cuddly koala exterior. Four participants referred to it as “cute” or “adorable,” and P4 said that it was like “a living creature.” It is possible that participants would have performed their touch gestures differently, or answered the evaluations differently, if the robot had a different appearance (e.g., a humanoid, an abstract design, or a scarier animal). Participants’ ratings on the three relevant questions of the robot acceptance survey showed they liked the robot’s presence, they found the robot to be non-threatening, and they were not afraid of it. These positive ratings could explain why participants expressed discomfort in hitting the robot. Future research could investigate how people touch and act toward touch-sensing robots with other appearances and form factors.

### 6.6 Gesture-Classification Approach

We asked participants to perform each touch gesture for a fixed duration of 5 s per trial. Within these 5 s, the user could do the gesture as many times as they wanted. Like the variability in exerted force intensity, these variations in gesture frequency likely made the gesture classification task more difficult. During a natural interaction between a user and the robot, some gestures might be conducted over time frames that are different from our data collection window, such as a user poking or hitting only once. While windowing is a common method for classifying time-series data, another method would be to use thresholding on the signals themselves, or their derivatives, to promptly identify the start and end of contact.

Nonetheless, our preliminary results suggest that the proposed touch-perception system can accurately detect social touch including the contacted body part, force intensity, and gesture. With an average accuracy of 74.1% (about eight times better than chance), our classifier performs comparably to other social-touch setups with more complex hardware and software, such as Keshmiri et al. (2020), even when identifying more force and gesture combinations. These results support our hypothesis that the rich time-series resistance data from our tactile sensors can capture both the gesture and force level conducted. Future researchers can further improve the presented data collection and analysis approaches for specific use cases.

### 6.7 Implications for Future Research

People enjoyed touching the soft, sensor-covered robot. Their impression of the robot was also significantly improved after interacting with it. Furthermore, multiple participants actively voiced displeasure when asked to hit the robot, with some opting to touch it in gentler ways. Altogether, these results lend credence to our hypothesis that users would find it appealing and engaging to interact with a touch-perceptive robot. It will be interesting to study how user impressions further evolve when a robot provides a response to their touches. Although we received initial recommendations from our participants, additional research will be needed to investigate the optimal ways for a robot to react to the various touch gestures it feels. Different responses may be more context appropriate to the input based on the robot’s role. For example, robots serving as caretakers, teachers, or animal companions might all need to react differently to an “energetic poking” gesture.

For this study, we covered NAO’s left arm with tactile sensors. As a next step, we want to extend sensor coverage across NAO’s entire body. We are now starting to digitize the sensor fabrication process, including designing additional sensors in drafting software, utilizing a laser cutter to create the layers, and swapping out our clothing iron for a mug press, as shown the supplementary fabrication video. These steps will streamline the sensor creation process and ensure even higher standards of reproducibility. We also would like to assist others in creating similar sensors for their own hard-bodied robot systems. Therefore, we plan to continue creating and sharing sensor patterns for the NAO robot in our database (Burns et al., 2022a).

Our next goal is total system integration. We plan to move the gesture classification from offline to real-time operation by establishing direct communication between the tactile sensors and the algorithm. This improvement will let us test how well our classification approach generalizes to completely new sensor data, and it will enable the robot to immediately react to detected gestures using sounds, lights, or movements. When the robot has the ability to move its body, we will need to account for any mechanical stimulation of the sensors caused by the robot’s own motion. We plan to train our system to predict self-touch artifacts generated by robot motions so that it can quickly and accurately identify external contacts that occur.

To date, there has been a glaring absence of social-touch perception in the current technology for human-robot interaction and socially assistive robotics. Conducting this study has proven to us that it is both possible and worthwhile to add tactile perception of both gesture and force intensities to social robots. Although the task of fully integrating an external system into an existing robot can be challenging, we believe that adding touch perception will enable robots to mimic the types of social touch interactions that occur so commonly between humans in everyday life, thereby providing users with more engaging and meaningful teaching, assistance, and companionship experiences.

## Data Availability

The datasets presented in this study can be found in online repositories. The sensor pattern collection is accessible at https://dx.doi.org/10.17617/3.6x. The user study dataset is accessible at https://dx.doi.org/10.17617/3.6w.

## Supplementary Material

Supplementary Video S1 A step-by-step video shows the entire fabrication process for our custom fabric-based tactile sensors.

Supplementary Video S2 A visual excerpt from the user study shows a participant performing a variety of gestures on the robot, as seen through the experimenter’s interface.

Supplementary Image 1 Box-and-whisker plots indicate the range of sensor resistance readings during participants’ gesture performances. From left to right, the plots evaluate gestures conducted on the hand, lower-arm, upper-arm, and shoulder sensors. From top to bottom, the gestures evaluated are hitting, poking, squeezing, stroking, and tickling. Within each plot, participants are ordered according to the median resistance value for the energetic gesture (low to high median resistance). Gentle trials are shown in a pale color directly next to the more darkly colored energetic trials of the same type.
