# Supplementary figures and images for: Endowing a NAO Robot With Practical Social-Touch Perception
Source: Front Robot AI. 2022 Apr 19;9:840335. doi: 10.3389/frobt.2022.840335 (PMC9061995; doi:10.3389/frobt.2022.840335)

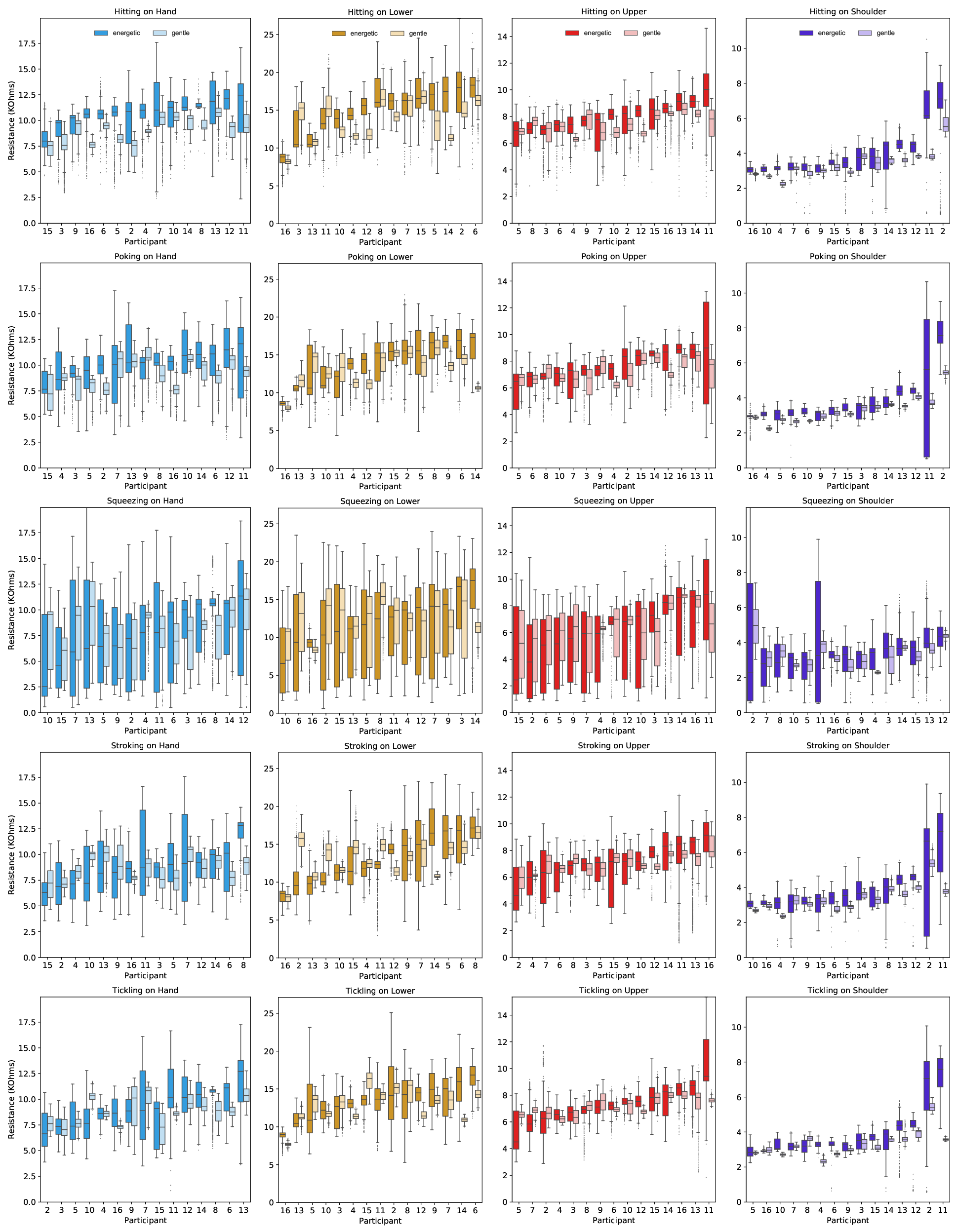

Supplement: Supplementary file 1 [file Image1.TIF]
